# The Diagnosis between Duodenal Ulcer and Gall Stone Disease

**Published:** 1907-03

**Authors:** 


### THE DIAGNOSIS BETWEEN DUODENAL ULCER AND GALL STONE DISEASE.

After considering this subject, Christopher Graham says:

That pain in cholecystitis is sudden and severe, usually has a wide field of radiation, comes with no regularity as to time, is rarely caused by food and as rarely eased by it, nor does the patient often trace his distress to it. There is no stomach history between the short sharp attacks; spasm of the diaphragm with dyspnea is common, vomiting and gas, if present, are so only during the colic, and the relief form eructation and vomiting is not so marked as in ulcer. Nausea and intense retching may be followed by vomiting of a small amount of thin, yellowish, bitter liquid mixed with mucus.

In duodenal ulcer pains comes in periods of attack lasting for days or weeks, is often sudden, may be severe, yet usually not that intense type of pain met in gallstones, but rather gnawing and burning in character. It may be irregular as to time of the separate attacks, but regular during the period of the stomach disturbance. The pain is clearly related to food, the intensity often modified by kind and quantity taken. Food eases for a time, the pain returning from 2 to 4 hours later. Hot drinks, soda and irrigation give relief. Spasm of the diaphragm is rarely seen except in some cases of perforation.

The chronic gallstone case, with impacted stone, ulceration and adhesions in which no jaundice appears and the stomach symptoms as gas, vomiting, burning distress, sour eructation, impaired appetite and dilation predominate, and the pain is moderate and follows food, will too often be diagnosticated ulcer; while the duodenal case, in whose early history we can elicit only irregular attacks of sudden, sharp, intense pain of peritonitis or acute spasm (and with no obstruction or hyperacidity) we do not have gas, vomiting or sour eructations, will as surely be mistaken for gallstones.

To the conceits of surgery we shall too often be obliged to leave the differentation of this class of cases and to its comprehensiveness the surety of relief.—Journal A. M. A., February 9, 1907.

